# An Atypical Presentation of Richter's Transformation in Chronic Lymphocytic Leukemia: Stroke-Like Onset With Multiorgan Involvement Including the Heart and Seminal Vesicles

**DOI:** 10.7759/cureus.116401

**Published:** 2026-09-17

**Authors:** Aysha Musthafa, Gayatri Gopan

**Affiliations:** 1 Medical Oncology, Sri Ramakrishna Ashrama Charitable Hospital, Thiruvananthapuram, IND

**Keywords:** chronic lymphocytic leukemia, extranodal lymphomas, fluorodeoxyglucose positron emission tomography, high-grade b-cell lymphoma, r-chop chemotherapy, richter's transformation

## Abstract

Chronic lymphocytic leukemia (CLL) is usually an indolent lymphoproliferative disorder. However, a small proportion of patients may develop Richter’s transformation (RT), in which CLL transforms into an aggressive lymphoma. This transformation is often associated with rapid clinical progression and extranodal disease.

Here, we describe a 72-year-old man diagnosed with CLL during evaluation for persistent cough and leukocytosis. Peripheral blood smear demonstrated smudge cells, and the diagnosis was confirmed by peripheral blood flow cytometry. As the patient was asymptomatic, he was managed with a watch-and-wait approach and regular follow-up. After nearly three years of observation, the patient presented with acute-onset slurring of speech and right-sided weakness, initially raising suspicion for a cerebrovascular event. Further imaging revealed multiple lesions involving the brain, lungs, stomach, and seminal vesicles, without lymph node involvement or hepatosplenomegaly. Fluorodeoxyglucose-positron emission tomography (FDG-PET) demonstrated widespread metabolically active disease. Histopathological examination of a CT-guided lung biopsy revealed high-grade B-cell lymphoma, raising suspicion for RT in the setting of known CLL.

The patient was treated with R-CHOP chemotherapy, and follow-up imaging showed a favorable radiological response.

This case highlights the atypical presentation of RT in patients with CLL who develop sudden clinical deterioration or unusual extranodal involvement, the role of tissue biopsy in establishing the diagnosis, and the challenges involved in its management.

## Introduction

Chronic lymphocytic leukemia (CLL) is a clonal proliferation of mature, CD5-positive B lymphocytes, diagnosed by peripheral blood flow cytometry demonstrating a characteristic immunophenotype, typically with an absolute B-lymphocyte count of ≥5 × 10⁹/L sustained over time. It is typically an indolent hematological malignancy, and the majority of patients remain asymptomatic for prolonged periods and are managed with a watch-and-wait approach [1].

A small proportion of patients, however, undergo Richter’s transformation (RT), defined as the evolution of CLL into a more aggressive lymphoma, most commonly diffuse large B-cell lymphoma (DLBCL), affecting approximately 2%-10% of patients with CLL [2]. The risk of transformation is not uniform among patients with CLL. Molecular and clinical features associated with a higher risk of RT include unmutated immunoglobulin heavy-chain variable region (IGHV) status, TP53 disruption, NOTCH1 mutations, and advanced disease at diagnosis. TP53 disruption and other adverse molecular features have also been associated with poor outcomes following transformation, contributing to the markedly worse survival observed in RT compared with progressive CLL.

Classically, RT presents with rapidly enlarging lymphadenopathy, B symptoms, and a rising lactate dehydrogenase (LDH) level, which may raise clinical suspicion for transformation [3]. However, atypical presentations are increasingly recognized and pose a distinct diagnostic challenge. Extranodal involvement, including gastrointestinal, pulmonary, and, less commonly, cardiac or genitourinary sites, as well as presentations mimicking unrelated acute conditions, can delay diagnosis and worsen outcomes [4]. Because early recognition and biopsy confirmation are essential for guiding timely treatment, unusual presentations of RT warrant particular clinical attention.

We report a case of RT in a patient with previously stable, early-stage CLL (Rai stage I) who presented with an acute neurological syndrome mimicking cerebrovascular disease and was subsequently found to have extensive multiorgan involvement. The disease involved the brain, lungs, stomach, seminal vesicles, and right atrium, representing an atypical pattern of extranodal disease. This case highlights the diagnostic pitfalls posed by an atypical presentation and emphasizes that RT should be suspected even in the absence of hepatosplenomegaly and lymphadenopathy. It also underscores the role of comprehensive metabolic imaging in defining disease extent and identifying an appropriate biopsy site [5]. Extranodal involvement may affect diverse organ systems, emphasizing the heterogeneous clinical spectrum of RT and the importance of whole-body assessment when transformation is suspected. RT is a clinicopathological diagnosis requiring tissue biopsy of a suspicious site, guided where possible by the most fluorodeoxyglucose (FDG)-avid lesion on positron emission tomography/computed tomography (PET/CT), to confirm large-cell transformation, as clinical suspicion alone is insufficient for diagnosis [6].

## Case presentation

A 72-year-old man was initially evaluated in 2022 for a persistent cough lasting one month. Routine blood investigations revealed persistent leukocytosis. Peripheral blood smear demonstrated smudge cells, and a diagnosis of CLL (Rai stage I) was confirmed by flow cytometry. Peripheral blood flow cytometry showed positivity for CD5 and CD23, with CD20 also positive. The total leukocyte count was approximately 50 × 10⁹/L, with an absolute lymphocyte count of approximately 30 × 10⁹/L.

As the patient was clinically asymptomatic, he was managed with a watch-and-wait approach and followed up with routine clinical and laboratory assessments.

In June 2025, the patient developed acute-onset slurring of speech and right-sided weakness, raising concern for an acute cerebrovascular event. Neurological examination was performed, and magnetic resonance imaging (MRI) of the brain revealed a well-defined, oval-shaped, intra-axial, T2/fluid-attenuated inversion recovery (FLAIR) ring-enhancing mass lesion in the left precentral gyrus of the frontotemporal lobe, involving Broca’s area, with perilesional edema. The lesion measured 18.2 × 21.5 × 13.7 mm and was suggestive of a neoplastic etiology. Further evaluation with FDG-PET/CT and contrast-enhanced CT (CECT) (Figure 1 and Figure 2) revealed multiple metabolically active lesions involving the brain, bilateral lungs, stomach, seminal vesicles, and right atrium, without hepatosplenomegaly or lymphadenopathy, suggesting a disseminated malignant process. Transthoracic echocardiography did not reveal evidence of thrombus, raising suspicion that the metabolically active right atrial lesion represented lymphomatous involvement rather than thrombus. Given the extensive multiorgan involvement and the intracranial mass lesion, the differential diagnoses included metastatic malignancy from an unknown primary site. Although CNS involvement can occur in aggressive lymphoma, an intracranial mass lesion is an uncommon presentation of transformed CLL.

**Figure 1:**
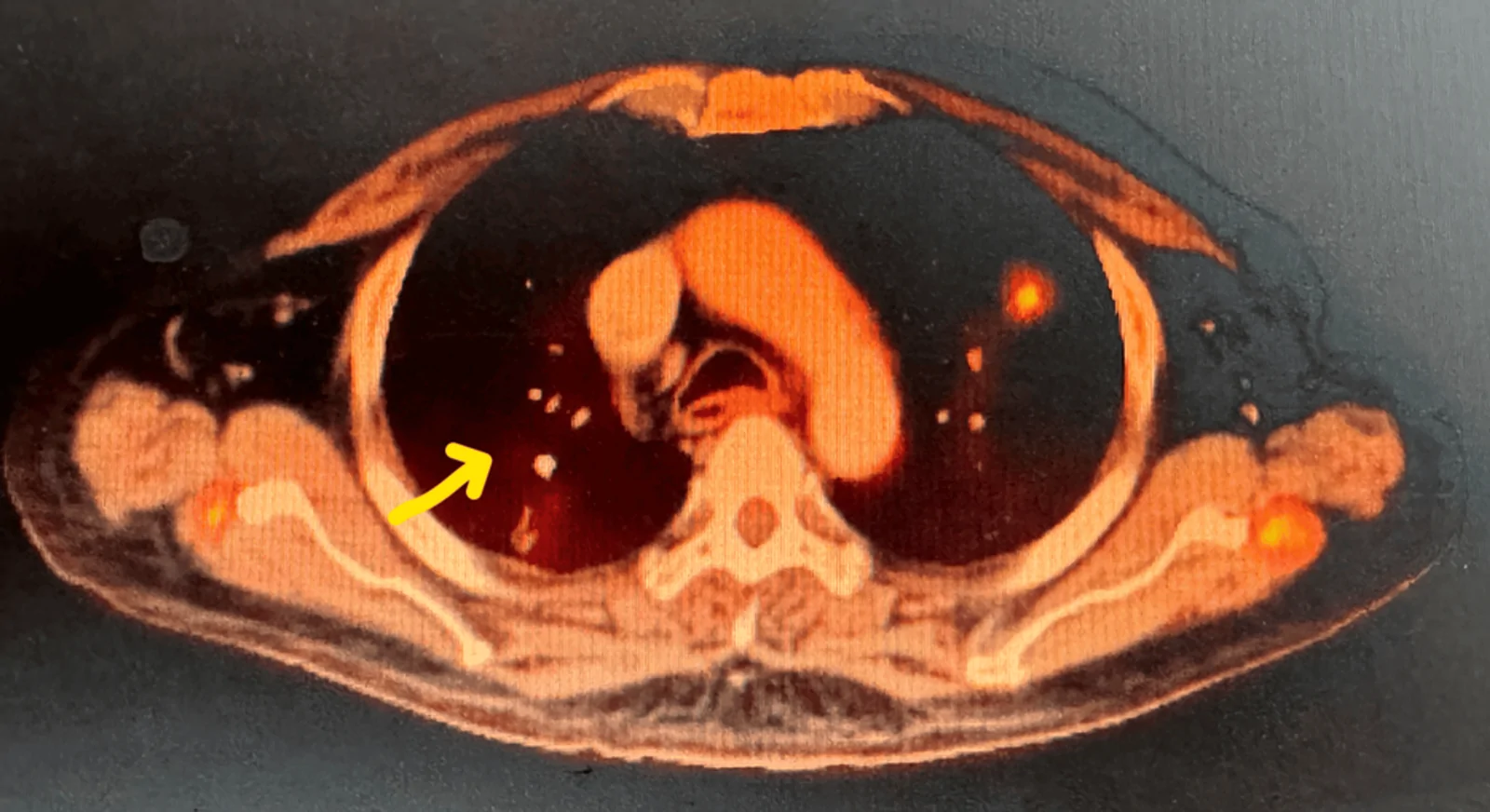
Axial fused FDG-PET/CT image showing metabolically active thoracic disease Axial fused FDG-PET/CT image demonstrated intense FDG uptake in the mediastinal lymph nodes and bilateral pulmonary lesions, indicative of widespread metabolically active disease. The imaging findings are suggestive of aggressive lymphomatous transformation consistent with Richter’s transformation. FDG: fluorodeoxyglucose, PET: positron emission tomography, CT: computed tomography.

**Figure 2:**
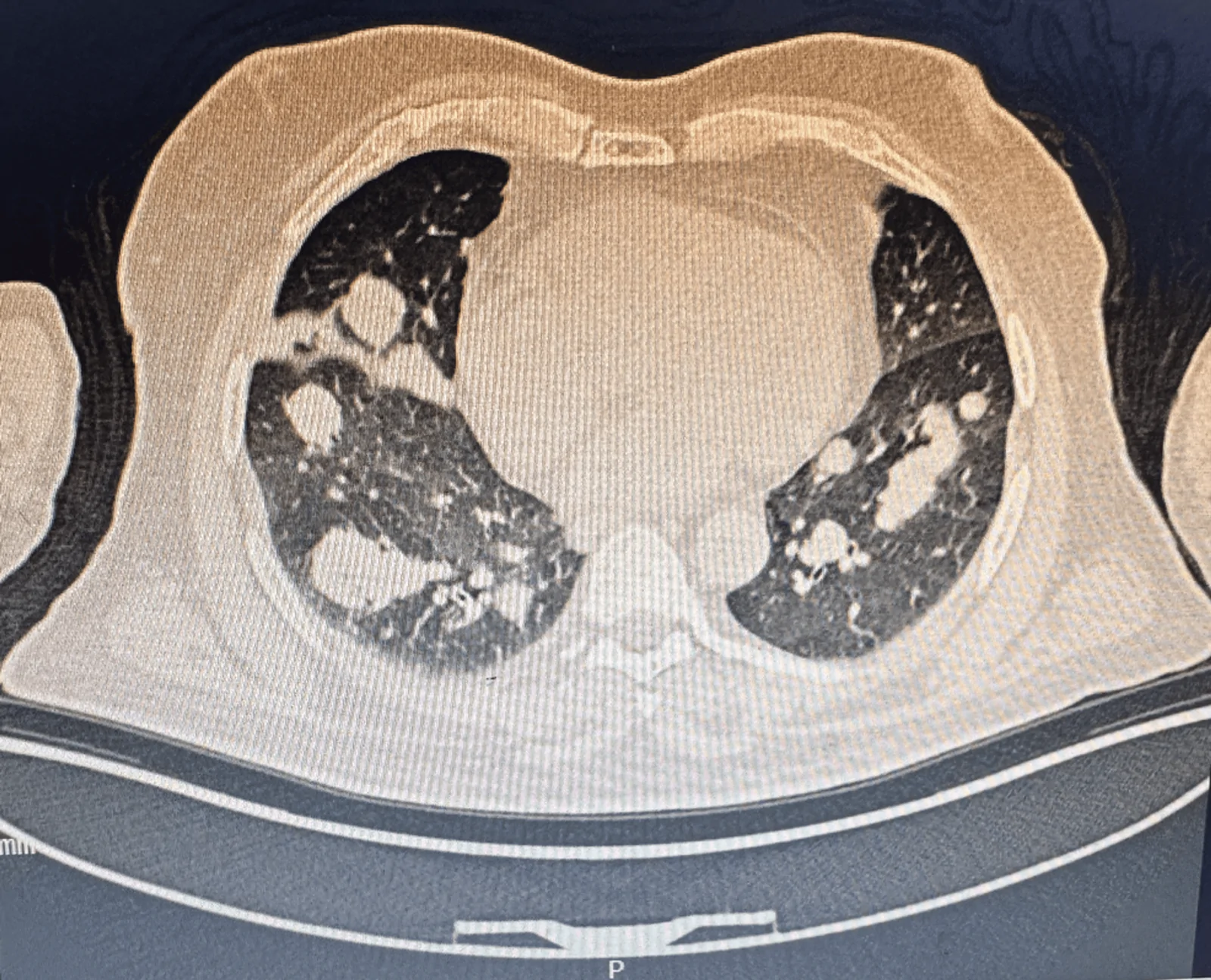
CECT scan of the thorax CECT of the thorax demonstrated multiple bilateral pulmonary nodules and masses corresponding to the metabolically active lesions seen on PET/CT. CECT: contrast-enhanced computed tomography, PET/CT: positron emission tomography/computed tomography.

An initial biopsy from the stomach (Table 1) revealed only ulceration and granulation tissue, without definitive evidence of malignancy. Given the persistent radiological abnormalities and the need for tissue diagnosis, further evaluation and biopsy of another accessible lesion were pursued. However, a CT-guided biopsy of a lung lesion (Figure 3) was suggestive of a lymphoproliferative lesion. Immunohistochemistry (Table 2) subsequently demonstrated strong, diffuse positivity for CD20, CD19, and leukocyte common antigen (LCA), with a high Ki-67 proliferative index of approximately 80%. The neoplastic cells were negative for TTF-1, CK7, AMACR, CD5, BCL2, BCL6, MUM1, cyclin D1, and ALK1, with focal positivity for CD10 and limited CD30 expression (~20%). Molecular testing showed no BCL2 or BCL6 rearrangements. Fluorescence in situ hybridization (FISH) testing for MYC, BCL2, and BCL6 rearrangements was not performed because of resource limitations; therefore, double-hit/triple-hit lymphoma could not be formally excluded. Similarly, clonal relatedness testing between the CLL and the high-grade B-cell lymphoma, such as IGHV sequencing, was not performed because of financial constraints. Therefore, a clonally unrelated de novo DLBCL cannot be entirely excluded, although the clinical context favored RT. The patient was diagnosed with high-grade B-cell lymphoma according to the WHO classification, with stage IV disease according to the Ann Arbor/Lugano staging system.

**Table 1:** Upper gastrointestinal endoscopic findings Upper gastrointestinal endoscopy revealed a depressed ulcer with raised margins and central necrosis in the proximal stomach along the greater curvature, suspicious for malignant involvement.

Parameter	Finding
Indication	Chronic lymphocytic leukemia
Scope	Olympus GIF H170
Premedication	10% lignocaine spray
Oral cavity	Normal
Esophagus	Normal
OG junction	37 cm from incisors
Fundus	Erythematous mucosa
Body of stomach	Depressed ulcer with raised margins measuring 2 cm, with a central necrotic area, identified along the greater curvature, approximately 5 cm below the gastroesophageal junction. Multiple biopsies were taken
Antrum	Normal
Pylorus	Normal
Duodenum (D1)	Few erosions at D1-D2 junction; biopsy taken
Duodenum (D2)	Normal
Endoscopic impression	Suspicious for malignant gastric ulcer; erosions at D1-D2 junction

**Figure 3:**
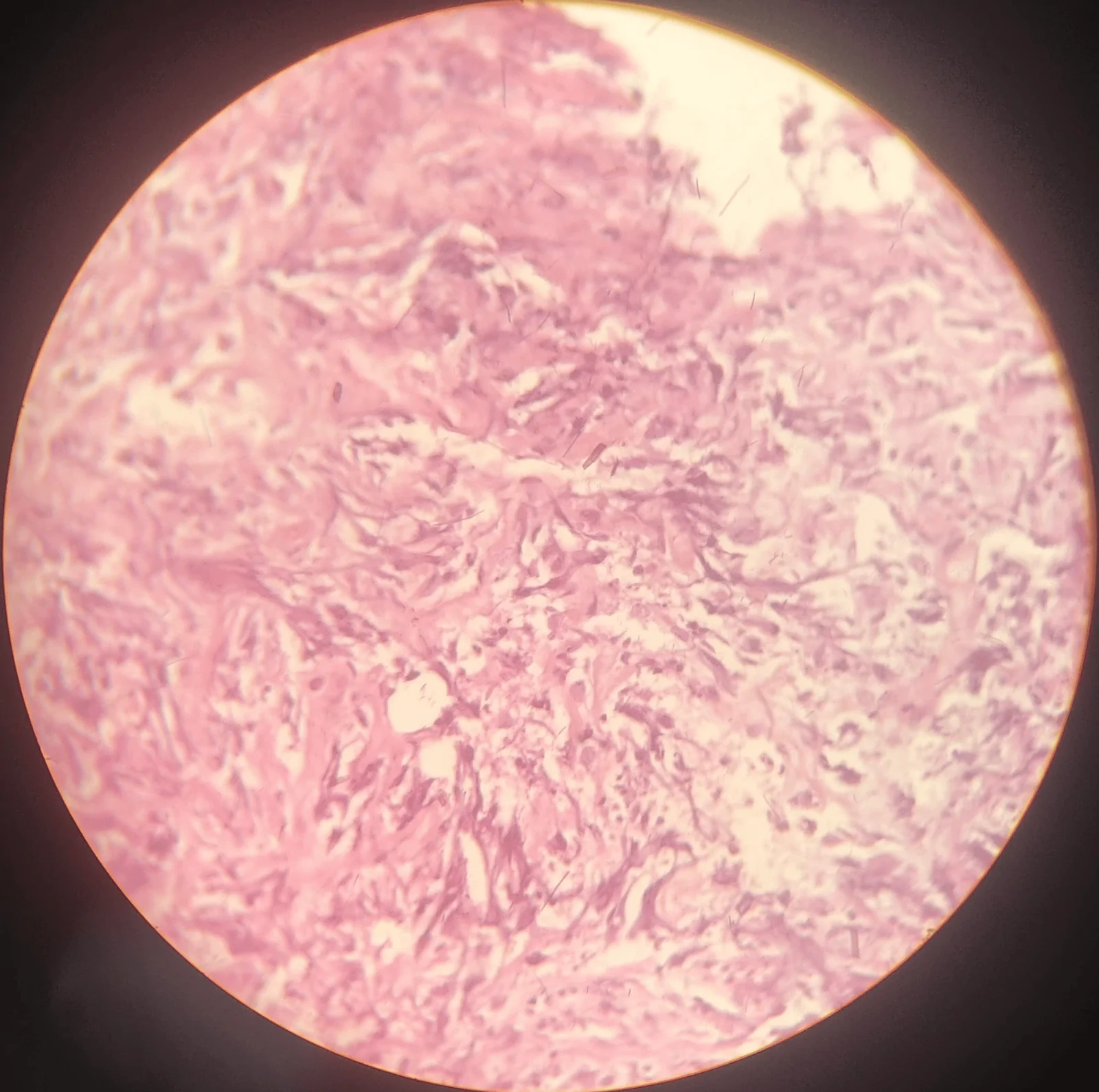
Histopathological features of Richter's transformation Hematoxylin and eosin-stained sections showed diffuse effacement of the normal tissue architecture by sheets of large atypical lymphoid cells, consistent with a high-grade lymphoid neoplasm in the setting of Richter’s transformation (H&E, ×10 and ×40).

**Table 2:** Immunohistochemistry profile of lung biopsy Immunohistochemistry demonstrated strong, diffuse positivity for CD20 and CD19, with a high Ki-67 proliferation index (80%), supporting the diagnosis of high-grade B-cell lymphoma consistent with Richter’s transformation.

Immunohistochemistry	
CD20	Strong diffuse positive
CD19	Positive
LCA (CD45)	Positive
Ki-67	80%
CD10	Positive (focal)
CD30	Few cells positive (20%)
CD3	Scanty cells positive
CD5	Negative
BCL2	Negative
BCL6	Negative
MUM1	Negative
Cyclin D1	Negative
TTF-1	Negative
AMACR	Negative
CK7	Negative (respiratory mucosa positive - internal control)
CD43	Negative
ALK-1	Negative
Overall impression	Immuno-profile suggestive of high-grade B-cell non-Hodgkin lymphoma, consistent with Richter's transformation

The brain lesion was treated locally with whole-brain radiotherapy. The patient received 30 Gy in 15 fractions but subsequently developed worsening breathlessness. Further imaging demonstrated progression of pulmonary involvement, and the patient required admission to the intensive care unit for respiratory support. He was subsequently started on R-CHOP chemotherapy, which was selected as the initial treatment regimen in view of the aggressive B-cell lymphoma phenotype and the clinical context of RT.

After completing six cycles of R-CHOP, follow-up imaging in October 2025 demonstrated a significant reduction in the pulmonary lesions, with the largest lesion now measuring 1.7 × 1.6 cm. There was also a reduction in cervical and mediastinal lymphadenopathy, with the largest lymph node measuring 7 × 4.8 mm in right level II, compared with the previous imaging performed in July 2025, indicating a favorable radiological response.

## Discussion

RT is a rare but clinically significant complication of CLL, most commonly manifesting as DLBCL and associated with poor prognosis [1,2]. Unlike the often indolent course of CLL, RT typically follows an aggressive clinical course and may involve multiple organ systems. Classical features include rapidly enlarging lymphadenopathy, B symptoms, and elevated LDH levels [3]. Molecular abnormalities, including TP53 disruption, NOTCH1 mutations, and unmutated IGHV status, have been associated with an increased risk of transformation.

In this case, the patient’s presentation with neurological symptoms mimicking an acute stroke represented an important diagnostic challenge. The subsequent identification of multisystem involvement, including the brain, lungs, stomach, seminal vesicles, and right atrium, without hepatosplenomegaly or lymphadenopathy, underscored the heterogeneous and atypical presentation of aggressive lymphoma in the setting of CLL. FDG-PET/CT and CECT (Figure 1 and Figure 2) helped identify metabolically active disease sites and guided the selection of a lung lesion for biopsy, which established the diagnosis of high-grade B-cell lymphoma [4]. The immunohistochemical profile (Table 2), including strong CD20 positivity and a high Ki-67 proliferative index, supported the diagnosis of an aggressive B-cell lymphoma consistent with RT.

Treatment of RT remains challenging, with no universally established standard regimen and generally poor outcomes despite aggressive therapy [5]. Therapeutic strategies increasingly include cellular therapies and novel immunotherapeutic approaches, including CAR-T cell therapy and bispecific antibodies, particularly in relapsed or refractory disease. In this case, R-CHOP was used as initial systemic therapy, with subsequent imaging demonstrating a favorable radiological response accompanied by clinical improvement.

International expert recommendations favor FDG-PET/CT in the workup of suspected RT, with biopsy of the most FDG-avid accessible site being prioritized [6]. Although molecular assessment of clonal relatedness between the underlying CLL and the transformed lymphoma can provide additional diagnostic and prognostic information, such testing is not routinely performed in all clinical settings and may be limited by availability and cost [7].

This case reinforces the need for a high index of suspicion for RT in patients with CLL who present with sudden or unusual symptoms, careful selection of biopsy sites to establish an accurate diagnosis, and a multidisciplinary approach to management. R-CHOP remains one of the commonly used chemoimmunotherapy approaches for RT with a DLBCL phenotype, although responses are frequently inadequate and novel therapeutic strategies are being investigated [8]. CNS involvement in RT is uncommon and remains poorly characterized, with much of the available literature consisting of case reports and small series [9]. In a recent Mayo Clinic retrospective study of 36 patients with CNS involvement by RT, 33.3% had parenchymal involvement, while 47.2% had concurrent systemic and CNS disease [9]. Patients with concurrent systemic and CNS involvement had a particularly poor prognosis, with a median overall survival of 3.1 months compared with 34.7 months among patients with isolated CNS disease [9]. In this case, the patient’s acute neurological presentation initially mimicked a cerebrovascular event and was subsequently found to be associated with a parenchymal brain lesion together with extensive systemic disease, highlighting the diagnostic challenge posed by CNS involvement. Early recognition and prompt tissue diagnosis are essential to facilitate timely treatment.

In a large single-center series of 204 patients with biopsy-confirmed RT, R-CHOP-like therapy was the most commonly used first-line regimen, although overall outcomes remained poor, with a median overall survival of 12 months [10]. In our patient, six cycles of R-CHOP resulted in a favorable radiological response; however, longer follow-up would be required to determine the durability of this response.

## Conclusions

RT is a rare but aggressive complication of CLL, and its unpredictable nature warrants consideration in patients presenting with new or rapidly progressive extranodal involvement. This case illustrates two diagnostic pitfalls that may delay recognition of RT. First, the patient’s acute neurological presentation initially mimicked a cerebrovascular event. In patients with CLL who develop acute neurological symptoms, CNS involvement by aggressive lymphoma should be considered, particularly when the clinical findings or neuroimaging are atypical for an acute stroke. Second, this patient demonstrated extensive multiorgan involvement, including the right atrium and seminal vesicles. This highlights the potential for unusual extranodal disease in RT and the value of comprehensive metabolic imaging with FDG-PET/CT when transformation is suspected.

Taken together, these findings suggest that unusual presentations of RT, whether neurological or involving multiple organ systems, should prompt comprehensive imaging and a low threshold for biopsy. This case underscores the importance of continued clinical surveillance in patients with CLL, including those with previously stable disease, and highlights the need to consider RT when new, unexplained clinical deterioration occurs.
